# AC-93253 iodide, a novel Src inhibitor, suppresses NSCLC progression by modulating multiple Src-related signaling pathways

**DOI:** 10.1186/s13045-017-0539-3

**Published:** 2017-11-13

**Authors:** Yi-Hua Lai, Sih-Yin Lin, Yu-Shan Wu, Huei-Wen Chen, Jeremy J. W. Chen

**Affiliations:** 1 Institute of Biomedical Sciences, National Chung Hsing University, No. 145, Xingda Rd., South Dist, Taichung, 40227 Taiwan, Republic of China; 2 Department of Chemistry, Tunghai University, Taichung, Taiwan; 3 Graduate Institute of Toxicology, National Taiwan University College of Medicine, Taipei, Taiwan; 4 Agricultural Biotechnology Center, National Chung Hsing University, Taichung, Taiwan; 5 Department of Medical Research, China Medical University Hospital, China Medical University, Taichung, Taiwan; 6 Department of Biotechnology, Asia University, Taichung, 41354 Taiwan

**Keywords:** Src, EGFR, NSCLC, AC-93253 iodide, Gefitinib

## Abstract

**Background:**

The tyrosine kinase Src is involved in the progression of many cancers. Moreover, inhibiting Src activity has been shown to obstruct several signaling pathways regulated by the EGFR. Thus, Src is a valuable target molecule in drug development. The purpose of this study was to identify compounds that directly or indirectly modulate Src to suppress lung cancer cell growth and motility and to investigate the molecular mechanisms underlying the effects of these compounds.

**Methods:**

Human non-small cell lung cancer (NSCLC) cell lines (PC9, PC9/gef, A549, and H1975) with different EGFR statuses were tested by cytotoxicity and proliferation assays after AC-93253 iodide treatment. Src and Src-related protein expression in AC-93253 iodide-treated PC9, PC9/gef, and A549 cells were assessed by western blotting. The effects of AC-93253 iodide on cancer cell colony formation, invasion, and migration were assessed in PC9 and PC9/gef cells. The synergistic effects of gefitinib and AC-93253 iodide were evaluated by combination index (CI)-isobologram analysis in gefitinib-resistant cell lines. The efficacy of AC-93253 iodide in vivo was determined using nude mice treated with either the compound or the vehicle.

**Results:**

Among the compounds, AC-93253 iodide exhibited the most potent dose-independent inhibitory effects on the activity of Src as well as on that of the Src-related proteins EGFR, STAT3, and FAK. Furthermore, AC-93253 iodide significantly suppressed cancer cell proliferation, colony formation, invasion, and migration in vitro and tumor growth in vivo. AC-93253 iodide sensitized tumor cells to gefitinib treatment regardless of whether the cells were gefitinib-sensitive (PC9) or resistant (H1975 and PC9/gef), indicating that it may exert synergistic effects when used in combination with established therapeutic agents. Our findings also suggested that the inhibitory effects of AC-93253 iodide on lung cancer progression may be attributable to its ability to modulate multiple proteins, including Src, PI3K, JNK, Paxillin, p130cas, MEK, ERK, and EGFR.

**Conclusions:**

Our data suggest that AC-93253 iodide inhibits NSCLC cell growth and motility by regulating multiple Src-related pathways. Our findings may facilitate the development of therapeutic strategies and anti-tumor drugs that may be useful for treating lung cancer in the future.

**Electronic supplementary material:**

The online version of this article (10.1186/s13045-017-0539-3) contains supplementary material, which is available to authorized users.

## Background

Non-small cell lung cancer (NSCLC) is one of the leading causes of cancer-related mortality worldwide [1], and the survival rate associated with NSCLC remains relatively low due to a lack of effective treatments for patients with advanced and metastatic disease as well as patients with recurrent disease [2]. Oncogenic driver mutations are often the cause of normal cell functional dysregulation. Most of these mutations occur in signal transduction-related kinases, including HER2, KRAS, AKT1, MEK, and EGFR, and they cause constitutive kinase activation, thereby inducing aberrant cancer cell growth and metastasis, which lead to poor prognoses and poor patient treatment responses [3, 4]. Hence, inhibiting tumor growth by targeting these molecules is essential for improving the prognoses of patients with NSCLC and increasing the efficacies of treatments for the disease [5].

In the past few years, targeted therapies intended to treat lung cancer patients with certain driver mutations have been developed. For example, the tyrosine kinase inhibitors (TKIs) gefitinib and erlotinib have been administered to patients with NSCLC with EGFR exon 19 deletions or L858R mutations [6]. Moreover, crizotinib has been associated with good responses when used to treat Asian patients with EML4-ALK fusion-positive NSCLC [7]. However, approximately 10% of patients with NSCLC display primary TKI resistance, the underlying mechanism of which is unknown. The results of a previous study indicated that EGFR T790M mutations can lead to TKI resistance and are positively associated with lung cancer recurrence [8]. Drug resistance is a critical issue with respect to cancer treatment; therefore, developing new therapeutic strategies or new targeted drugs, including second-generation TKIs, exemplified by afatinib [9], and third-generation TKIs, including AZD9291 (osimertinib) [10], has become a main objective of the current studies focusing on cancer treatment.

Src is a proto-oncogene that encodes a tyrosine kinase, and it is known to participate in many signaling pathways, including the focal adhesion kinase (FAK), phosphatidylinositol 3-kinase (PI3K), and signal transducer and activator of transcription 3 (STAT3) pathways [11, 12]. Since Src activation ultimately promotes cancer cell survival, proliferation, invasion and tumoural angiogenesis, and Src phosphorylation and expression are closely associated with cancer progression. Moreover, a previous clinical investigation showed that patients with NSCLC with high Src activity or expression levels had a poor prognosis [13]. Furthermore, another study showed that upregulation of Src kinase activity and protein expression levels occurred in approximately 50% of patients with colon, liver, lung, prostate, or breast cancers [14]. Thus, the results of these studies indicate that Src may be a target in lung cancer treatment [15].

Because of the crosstalk between Src and EGFR, inhibiting Src may improve NSCLC treatment [13]. Thus, several Src inhibitors, including dasatinib (BMS-354825), bosutinib (SKI-606), and saracatinib (AZD-0530), have been developed, and their effectiveness against solid tumors has been evaluated in clinical trials [16]. Previous studies have shown that Src kinase inhibitors can induce NSCLC cell apoptosis and inhibit angiogenesis and, depending on the EGFR mutation status, EGFR tyrosine kinase activation [17, 18]. Furthermore, a previous study showed that combination therapy with Src inhibitors and gefitinib can enhance the effects of TKIs on EGFR and STAT3 [19].

To improve the efficacy of lung cancer treatments, we aimed to identify the compounds that can disrupt Src-EGFR cooperation and are less dependent on EGFR status than other compounds. In this study, using molecular docking and the LOPAC compound library, we determined that AC-93253 iodide (PubChem CID: 16078948) is a candidate Src-EGFR crosstalk inhibitor. We also investigated the functional mechanism underlying the ability of AC-93253 iodide to suppress lung cancer progression using in vitro and in vivo approaches. Our findings may facilitate the development of new anti-cancer drugs and therapeutic strategies useful for the treatment of lung cancer in the future.

## Methods

### Cell culture and drug treatment

The human lung adenocarcinoma cell lines A549 (ATCC CCL-185) and H1975 (ATCC CRL-5908), the human bronchial alveolar carcinoma cell line H358 (ATCC CRL-5807), and the human bronchial epithelial cell line BEAS2B (ATCC CRL-9609) were purchased from the American Type Culture Collection (ATCC, Manassas, VA, USA). The human lung adenocarcinoma cell lines PC9 and PC9/gef were kindly provided by Dr. Chih-Hsin Yang, NTU Hospital. These cell lines were cultured in RPMI-1640 media (Gibco, Carlsbad, CA, USA) supplemented with 10% fetal bovine serum (FBS; Gibco-Invitrogen) and 1% penicillin/streptomycin (Gibco) at 37 °C in a humidified atmosphere of 5% CO_2_. AC-93253 iodide was purchased from Sigma–Aldrich Chemical Co. (St. Louis, MO, USA) and was prepared in dimethyl sulphoxide (DMSO) as a stock solution with a concentration of 100 mM.

### Western blotting and real-time reverse transcription PCR (RT-PCR) analysis

Western blotting was performed to assess protein phosphorylation and expression levels in lung cancer cells treated with AC-93253 iodide as described previously [20]. The EGFR, STAT3 (F-2), PI3K, phospho-MEK1/2 (Ser 218/Ser 222), MEK, phospho-ERK (Tyr204), ERK, Paxillin, and p130cas were purchased from Santa Cruz Biotechnology, Inc. (Dallas, TX, USA). Phospho-Src (pY418), phospho-FAK (Tyr576), and FAK were purchased from Invitrogen (Carlsbad, CA, USA). Phospho-EGFR (Tyr1068), phospho-STAT3 (Tyr 705), phospho-PI3K (Tyr458), phospho-SAPK/Jun N-terminal kinase (JNK) (Thr183/Tyr185), SAPK/JNK, phospho-Paxillin (Tyr118), and phospho-p130cas (Tyr410) were purchased from Cell Signaling Technology (Beverly, MA, USA), and the primary antibody to Src was produced in our laboratory (ATCC CRL-2651). GAPDH (Upstate Biotechnology, Lake Placid, NY, USA) was used as a loading control. The mRNA expression levels of Src and related genes were detected using a real-time PCR machine (ABI prism 7300 Sequence Detection System, Applied Biosystems, Carlsbad, CA, USA) and SYBR Green Reagent (Roche, Basel, Switzerland). TATA-box binding protein (TBP) was used as an internal control (GenBank X54993). Details regarding the procedures and calculations used to perform the experiments are provided elsewhere [21].

### Cell viability and proliferation assays

PrestoBlue cell viability reagent (Invitrogen) was used according to the manufacturer’s instructions to evaluate the effects of AC-93253 iodide on cytotoxicity and cell proliferation. After the cells were treated with AC-93253 iodide at different concentrations or for different times, the designated volume of PrestoBlue reagent was added to each culture well to react with the cells, and then the absorbance was measured at 570 nm (with 600 nm as the reference) using a Victor^3^ spectrophotometer (Perkin-Elmer, Santa Clara, CA, USA).

### Colony formation assay

For the anchorage-dependent growth assay, 500 cells were seeded in a culture dish containing culture media and drug solution. After 7–10 days, the cells were washed with 1×PBS and fixed with methanol. The fixed cells were subsequently stained with 0.05% crystal violet. For the anchorage-independent growth assay, six-well plates were precoated with 2 ml of 0.7% agarose in RPMI supplemented with 10% FBS. Then, cells were seeded in the wells in 0.35% agarose/RPMI with 10% FBS at a density of 1 × 10^3^ cells per well. After the agar solidified, the cells were treated with AC-93253 iodide. The plates were subsequently incubated for 2 weeks before being stained with 0.5 mg/ml p-iodonitrotetrazolium violet. Colonies with a diameter greater than 0.2 mm were counted using an inverted microscope. Details regarding the methods used to perform this procedure are provided elsewhere [19].

### Migration and invasion assay

A transwell apparatus with a polycarbonate membrane (8-μm pore size, 6.5-mm diameter; Corning Costar Corporation, MA, USA) coated or not with Matrigel (2.5 mg/ml; BD Biosciences, San Jose, CA, USA) was used for transwell migration and invasion assays as described previously [21]. The upper wells were filled with serum-free medium containing the drug and the cells (5 × 10^3^ or 2 × 10^4^ cells per well), and the lower wells were filled with the same medium supplemented with 10% FBS. After 14 h (migration) or 24 h (invasion) of incubation, the cells in the upper wells and on the membrane were swabbed with a Q-tip, fixed with methanol, and stained with 10% Giemsa solution (Sigma Chemical). The cells that were attached to the lower surface of the polycarbonate filter were counted using a light microscope (magnification, ×200).

### Tumourigenesis assay

Tumors were induced in nude mice according to a previously described protocol [19]. Five-week-old severe combined immunodeficiency (SCID) nude mice were purchased from the National Laboratory Animal Center (NLAC, Taipei, Taiwan). A total of 2 × 10^6^ live PC9/gef cells were subcutaneously injected into the nude mice. Tumor volumes were measured every 3 days until they reached an average size of 50 mm^3^. To evaluate the tumor suppressive effects of AC-93253 iodide, we separated the mice into two groups, one treated with 0.1% DMSO and the other with 0.25 mg/kg of AC-93253 iodide. The animals in the former group were injected with 100 μl of PBS with 0.1% DMSO daily, whereas the animals in the latter group were injected with AC-93253 iodide. After 4–5 weeks, the mice were sacrificed using CO_2_, and their tumor volumes were estimated from caliper-measured lengths (a) and widths (b) using the following formula: *V* = 0.4 × *ab* ^2^ [22]. The mouse experiments were approved by the Institutional Animal Care and Use Committee of National Chung Hsing University.

### Protein degradation experiments and ubiquitination assays

AC-93253 iodide (0.1 μM) and/or cycloheximide (CHX, 2.5 μg/ml) (Sigma-Aldrich, St. Louis, MO, USA), a protein synthesis inhibitor, was employed to treat 3.5 × 10^5^ lung cancer PC9 cells. After 4, 8, and 12 h, we extracted the cell lysates and measured the expression levels of specific proteins by western blot analysis. In addition, to determine whether protein degradation occurred through a ubiquitination pathway, we analyzed the lysates of cells treated with 100 nM AC-93253 iodide and/or 50 nM MG132 (Sigma-Aldrich), a proteasome inhibitor, for 72 h by western blotting. In ubiquitination assays, PC9 cells treated with AC-93253 iodide (0.1 μM) for 72 h were lysed in lysis buffer and incubated with protein G Dynabeads (Invitrogen) to remove non-specifically bound proteins. After immunoprecipitation with the designated antibodies and protein G Dynabeads, the immunoprecipitated complexes were washed, separated by SDS-PAGE, and detected by immunoblotting with an anti-ubiquitin antibody (Sigma-Aldrich) as described previously [23].

### Drug synergy analysis

To investigate the synergic effects of AC-93253 iodide and gefitinib on lung cancer cells in vitro, we analyzed the data from the A549, PC9/gef, and H1975 cell proliferation assays with CalcuSyn software (Biosoft, Cambridge, UK) and the combination index (CI)-isobologram equation, as described previously [24]. CI < 1, CI = 1, and CI > 1 stand for the synergistic, additive, and antagonistic effects of the indicated compounds, respectively. In addition, immunoblotting was performed to investigate the effects of AC-93253 iodide, gefitinib, and combination treatment on Src-related proteins.

### Statistical analysis

All the experiments were performed at least in triplicate, and the results are presented as the mean ± standard deviation. Either *t* tests or ANOVA (Excel; Microsoft) were performed to determine the significance of the differences between groups. *P* values < 0.05 were considered statistically significant.

## Results

### Virtual screening of potential candidate compounds from the LOPAC library

Src activity is determined by its phosphorylation state as well as by protein–protein interactions on its SH2 and SH3 domains [25]. The phosphorylation occurs and the protein interactions initiate at tyrosine 418 [26]. It is possible to inhibit Src expression and prevent lung cancer progression by regulating the activities that occur at the site. The structures of the chemical compounds found in the LOPAC library, which comprises 1280 drugs, were docked into the Src tyrosine 418 site by the LibDock protocol of Discovery Studio v3.5, and the LibDock score and interaction force were calculated based on the docking poses of the compounds. The interaction force was adopted as the screening criterion to identify candidate Src-modulating compounds. We ultimately chose the 15 compounds predicted to have the strongest interactions with Src, as determined by the virtual screening process, as candidate compounds, which we labeled L1 to L15 (Additional file 1: Table S1). These candidate compounds were then subjected to further screening in subsequent biological analyses.

**Fig. 1:**
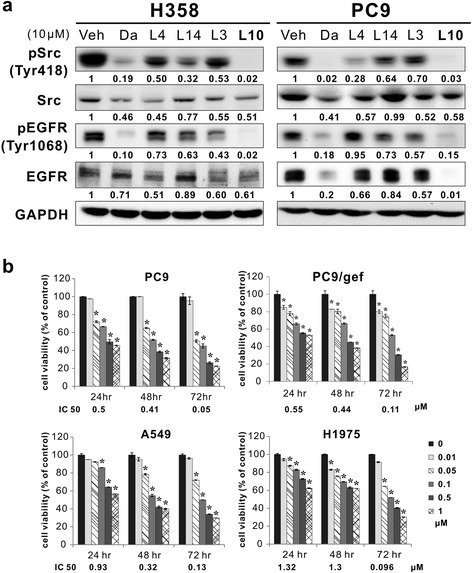
Effects of the candidate compounds on Src and EGFR expression and cell viability in different cell lines. **a** Src and EGFR expression and phosphorylation in H358 and PC9 cells treated with the candidate compounds for 24 h and analyzed by western blotting. Veh (vehicle) represents 0.1% DMSO; Da represents dasatinib, a positive control. GAPDH served as a loading control. Protein expression was quantified by ImageJ software (NIH), and the results are shown directly below the gel graph. **b** AC-93253 iodide cytotoxicity determined by cell viability assays in NSCLC cell lines with different EGFR statuses. The results are presented as percentages of the vehicle control (0 μM, 0.1% DMSO). The IC_50_ of the designated time point is indicated at the bottom of each bar chart. Each experiment was performed independently and in triplicate. *P < 0.05 compared with the vehicle control

During the initial screening, the lung cancer PC9 cell line was treated with candidate compounds at a concentration of 10 μM for 24 h, after which the cell lysates were used to investigate Src phosphorylation. Dasatinib was used as a positive control. The results of the experiment showed that L1, L3, L4, L10, L13, and L14 could inhibit Src activity (Additional file 1: Figure S1). Among these compounds, L3, L4, L10, and L14 were selected for additional experiments, in which their inhibitory effects on Src and EGFR activity in the H358 and PC9 cell lines were assessed. The results of those experiments showed that L10 could significantly suppress Src and EGFR phosphorylation in both cell lines (Fig. 1a) and that L10 exhibited moderate inhibitory effects on Src expression in both cell lines and significant inhibitory effects on EGFR expression in the PC9 cell line. Thus, compound L10, i.e., AC-93253 iodide, was selected for subsequent experiments intended to investigate the mechanisms underlying its inhibitory effects on the phosphorylation and expression of Src as well as those of related signaling effectors essential for tumor cell growth and motility.

### Cytotoxic effects of AC-93253 iodide on cancerous and noncancerous cells

To determine the optimal concentrations at which AC-93253 iodide should be administered in subsequent experiments, we performed cell viability assays, in which we assessed PC9 (EGFR^exon19 del^; gefitinib-sensitive), PC9/gef (EGFR^exon19 del;^ gefitinib-resistant), A549 (EGFR^wild-type^; gefitinib-resistant), and H1975 (EGFR^L858R+T790M^; gefitinib-resistant) lung adenocarcinoma cell viability over 24, 48, and 72 h of exposure to AC-93253 iodide. The results of the assay showed that AC-93253 iodide could inhibit cell survival in these four cell lines in a dose-dependent manner. Specifically, the results of the experiments showed that 0.05 μΜ AC-93253 iodide induced cell death in approximately 20–30% of gefitinib-resistant cells (PC9/gef, A549, and H1975) and that AC-93253 iodide concentrations > 0.1 μΜ induced cell death in > 50% of the indicated cell lines over 72 h. The IC_50_ for the indicated treatment time is shown in Fig. 1b. The IC_50_ values decreased by 10- to 100-fold on a nanomolar scale in a time-dependent manner. The IC_50_ values for non-tumoural BEAS2B cells were 9.07 μΜ over 24 h, 1.5 μΜ over 48 h, and 0.85 μΜ over 72 h and were higher than the values for tumor cells over the same time periods. The results of the cytotoxicity assays showed that AC-93253 iodide is relatively less toxic to normal cells than to cancer cells (Additional file 1: Figure S2).

### AC-93253 iodide suppresses the phosphorylation and expression of Src as well as that of Src downstream proteins

**Fig. 2:**
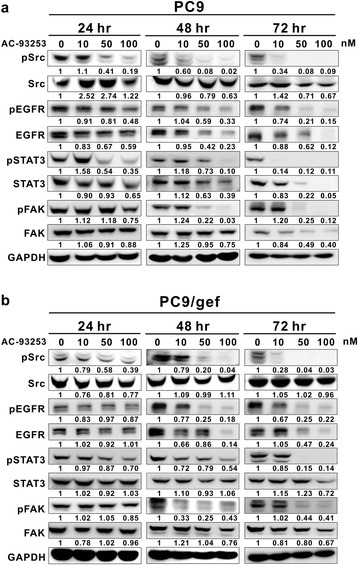
Effects of AC-93253 iodide on the expression and phosphorylation of Src and related proteins. Lung cancer PC9 cells (**a**) and PC9/gef cells (**b**) were treated with the designated concentrations of AC-93253 iodide for 24, 48, and 72 h. Protein expression was quantified by ImageJ software (NIH), and the results are shown directly below the gel graph. 0 nM: 0.1% DMSO. The Src, EGFR, STAT3, and FAK expression and phosphorylation levels were measured by immunoblot analysis using the indicated antibodies. GAPDH was used as an internal control. Each experiment was performed independently and was repeated three times

To compare the inhibitory effects of AC-93253 iodide on signaling pathways and cell functions in the different cell lines in vitro, based on the results of cell viability at 72 h, a range of concentrations comprising the IC_50_ and lower doses was used to perform subsequent experiments. After the cytotoxicity assays, the PC9 and PC9/gef cell lines were treated with AC-93253 iodide at concentrations of 10, 50, and 100 nM for 24, 48, and 72 h. The western blotting results showed that pSrc, pEGFR, EGFR, pSTAT3, STAT3, pFAK, and FAK expression levels decreased significantly in a dose- and time-dependent manner, whereas Src expression levels decreased slightly in PC9 cells (Fig. 2a). Interestingly, AC-93253 iodide exerted somewhat different inhibitory effects on these proteins in the PC9/gef cell line than in the PC9 cell line. pSrc, pEGFR, EGFR, pSTAT3, and pFAK expression levels decreased significantly in a dose- and time-dependent manner in PC9/gef cells; however, Src, STAT3, and FAK expression levels decreased only slightly (Fig. 2b). In addition, when A549 cells were treated with higher concentrations of AC-93253 iodide, Src phosphorylation, but not total protein expression, decreased (Additional file 1: Figure S3).

### AC-93253 iodide inhibits cancer cell proliferation and colony formation in vitro and tumor growth in vivo

**Fig. 3:**
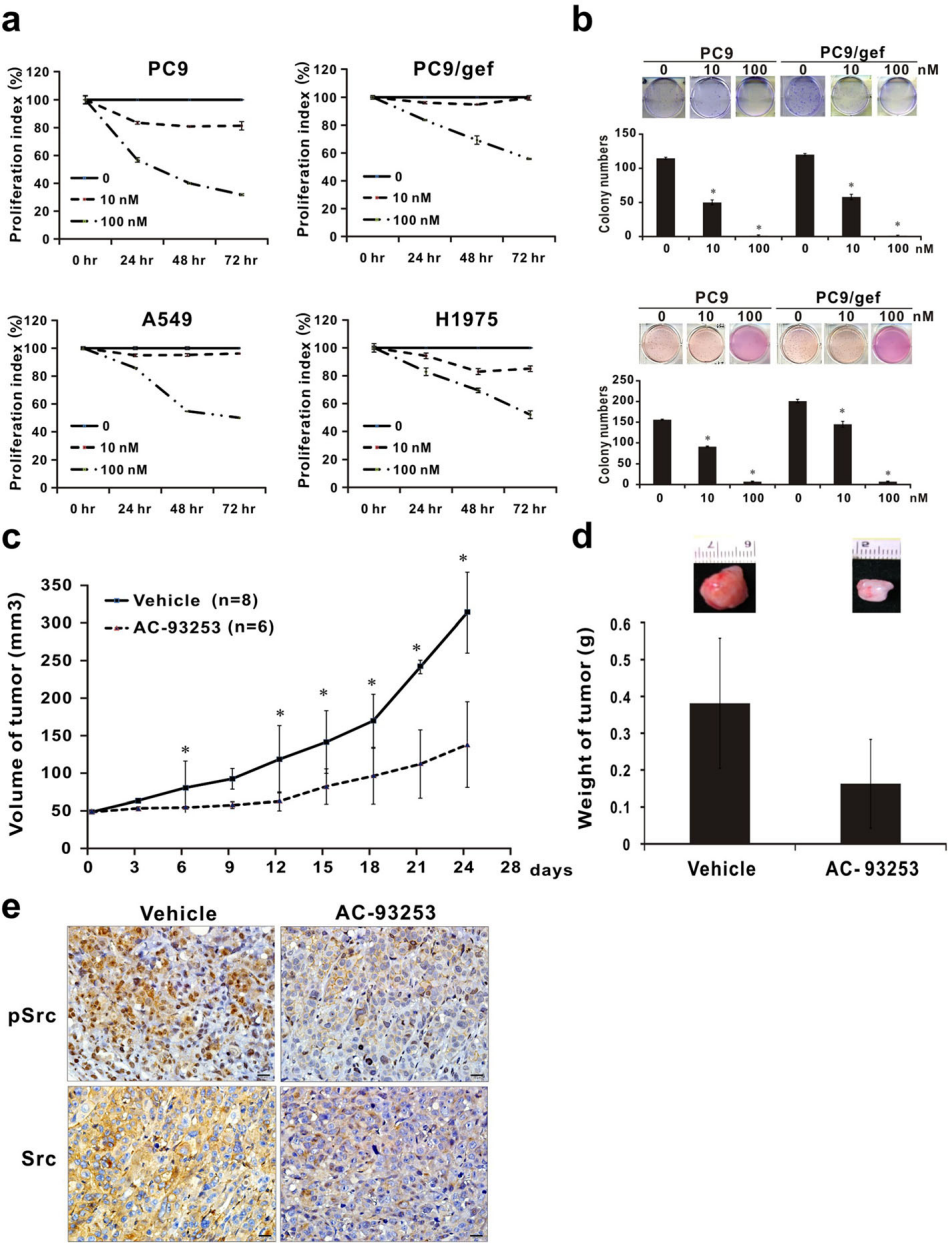
Suppressive effects of AC-93253 iodide on tumor cell growth. **a** AC-93253 iodide reduced proliferation in PC9, PC9/gef, A549, and H1975 cells as determined by PrestoBlue cell viability assays at the indicated time points. **b** AC-93253 iodide inhibited clonogenicity, as determined by colony formation assay. Upper panel: anchorage-dependent cell growth, colonies with diameters ≥ 0.3 mm were counted; lower panel: anchorage-independent cell growth, colony diameter ≥ 0.5 mm. Each experiment was performed independently and in triplicate; 0 nM: 0.1% DMSO. **c** Tumourigenesis assay. The indicated number of live PC9/gef cells was subcutaneously injected into mice divided into vehicle-treated (*n* = 8) and drug-treated groups (*n* = 6). Tumor volumes were measured every 3–4 days. **d** AC-93253 iodide decreased tumor weights, which were presented as the mean ± standard deviation. **e** pSrc and Src expression levels and distributions in murine tumor tissues were determined by immunohistochemical staining and observed using a light microscope (×400 magnification). Vehicle represents 0.1% DMSO, and AC-93253 represents 0.25 mg/kg of the compound. The scale bars represent 20 μm. *P < 0.05 compared with the vehicle control (0 nM, 0.1% DMSO)

To evaluate the anti-cancer effects of AC-93253 iodide, we performed tumor cell growth experiments in vitro and in vivo. We found that AC-93253 iodide significantly suppressed PC9, PC9/gef, A549, and H1975 cancer cell proliferation (Fig. 3a) and that AC-93253 iodide also inhibited anchorage-dependent and anchorage-independent cell colony formation in gefitinib-sensitive (PC9) and gefitinib-resistant (PC9/gef) cells even at a low concentration (Fig. 3b). We noted similar results in the A549 and CL1-5 lung cancer cell lines (Additional file 1: Figures S4 and S5).

To examine the impact of AC-93253 iodide on tumor growth in vivo, we injected PC9/gef cells subcutaneously into SCID mice. After their tumors reached the indicated volume, the mice were randomly assigned to a group treated with AC-93253 iodide (p.o., 0.25 mg/kg/day) or a control group and were injected intraperitoneally with the compound or the vehicle. Tumor volumes were measured every 3 days. The mean size and weight of the tumors in the former group were 26 mm^3^ (95% CI 7–48 mm^3^) and 182 mg, respectively, whereas the mean size and weight of the tumors in the latter group were 265 mm^3^ (95% CI 205–321 mm^3^) and 384 mg, respectively (Fig. 3c, d). Immunohistochemical staining demonstrated that pSrc and Src expression levels in the treated mice were significantly lower than in the control mice (Fig. 3e).

### AC-93253 iodide inhibits cancer cell motility and has synergistic effects when used in combination with other agents

**Fig. 4:**
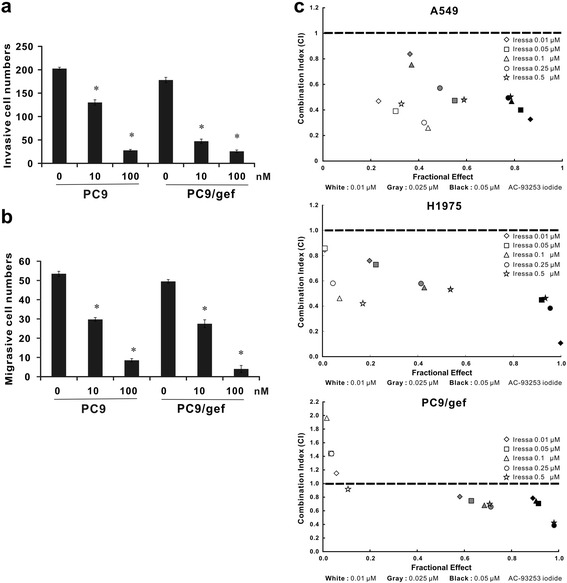
The impact of AC-93253 iodide on cell motility and drug synergism. **a** Inhibitory effects of AC-93253 iodide on cancer cell invasion as determined by the transwell assay with Matrigel. **b** Inhibitory effects of AC-93253 iodide on cancer cell migration as determined by the transwell assay without Matrigel. **c** Synergistic effects of AC-93253 iodide and gefitinib on gefitinib-resistant lung adenocarcinoma cells determined by cell viability assay. The indicated combinations of AC-93253 iodide and gefitinib were used to treat lung adenocarcinoma A549, PC9/gef, and H1975 cells for 72 h. The CI was calculated using the data and CalcuSyn software. Each experiment was performed independently and was repeated three times. *P < 0.05 compared with control (0 nM; 0.1% DMSO)

It is known that Src phosphorylation and expression can enhance cancer cell migration and invasion [27]. To investigate the effects of AC-93253 iodide on cancer cell motility, we treated PC9 and PC9/gef cells with various concentrations of AC-93253 iodide, which significantly inhibited cancer cell migration and invasion ability in treated cells compared with vehicle control cells (Fig. 4a, b). In addition, we treated the gefitinib-resistant lung adenocarcinoma cell lines A549, PC9/gef, and H1975 with various combinations of AC-93253 iodide and gefitinib for 72 h and then evaluated the combined effects of the two compounds on the cells. The results of the CI-isobologram analysis showed that AC-93253 iodide and gefitinib had synergistic effects on the A549 (CI 0.259~0.838), PC9/gef (CI 0.384~0.95), and H1975 cell lines (CI: 0.107~0.858) (Additional file 1: Tables S2–S4). Specifically, the combination of 0.01, 0.025, or 0.05 μM AC-93253 iodide and low-dose gefitinib (0.01 or 0.05 μM) had synergistic effects on the A549 and H1975 cell lines (Fig. 4c, upper and middle panels). Furthermore, the combination of 0.025 or 0.05 μM AC-93253 iodide and gefitinib rendered the PC9/gef cell line more sensitive to gefitinib, even when it was administered at concentrations as low as 0.01 μM (Fig. 4c, lower panel). Taken together, these results indicated that 0.025 μM and 0.05 μM AC-93253 iodide could sensitize A549, PC9/gef, and H1975 lung cancer cells to a wide range of gefitinib concentrations. To elucidate the possible mechanisms of synergy, gefitinib-resistant lung adenocarcinoma cell lines were treated with different combinations of AC-93253 iodide and gefitinib for 72 h and then analyzed by western blotting for Src-related proteins. Combination treatments could significantly suppress the phosphorylation and expression of EGFR, STAT3, and/or Src, especially at the 50 nM AC-93253 iodide concentration (Additional file 1: Figure S6). These data confirmed that the combined use of these two agents inhibited not only gefitinib-resistant cancer cell growth but also Src-related signaling pathways.

### Effects of AC-93253 iodide on the expression of proteins downstream from Src

**Fig. 5:**
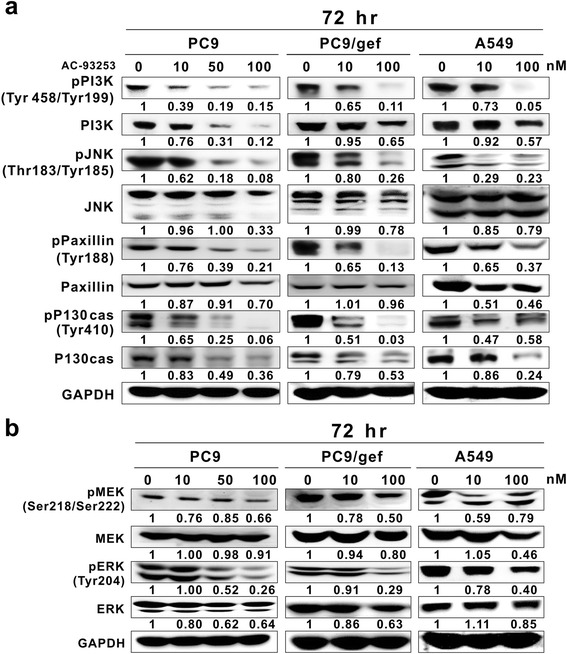
Expression of Src-related proteins in lung cancer cell lines treated with AC-93253 iodide. Lung cancer PC9, PC9/gef, and A549 cells were treated with AC-93253 iodide at the indicated concentrations for 72 h and then analyzed on western blots; 0 nM represents 0.1% DMSO. GAPDH was the loading control. **a** PI3K, JNK, Paxillin, and p130cas expression and phosphorylation levels. **b** MEK and ERK expression and phosphorylation levels. Protein expression was quantified by ImageJ software (NIH), and the results are shown directly below the gel graph. Each experiment was performed independently and in triplicate

Src activity can affect the expression of many downstream proteins, including STAT3, PI3K, JNK, Paxillin, p130cas, MEK, and ERK [28]; thus, we investigated whether AC-93253 iodide influences the expression of any of these proteins. We found that PI3K, JNK, Paxillin, and p130cas phosphorylation levels decreased significantly in conjunction with increases in AC-92353 iodide concentrations in both gefitinib-sensitive (PC9) and gefitinib-resistant cells (PC9/gef and A549) (Fig. 5a). However, the expression levels of these four proteins varied among the cell lines. Except for the dose-dependent inhibition of PI3K and p130cas protein levels in PC9 cells and the lack of effect on JNK in A549 cells, AC-93253 iodide had similar inhibitory effects on the phosphorylation and expression of these four proteins in the three cell lines, with the strongest effect found at 100 nM. Furthermore, AC-93253 iodide decreased MEK and ERK phosphorylation levels in either a dose-dependent manner from 0 to 100 nM or only at 100 nM, depending on the cell line (Fig. 5b). However, AC-93253 iodide induced only slight or non-significant changes in MEK and ERK total protein expression.

### Effects of AC-93253 iodide on protein degradation and transcription

**Fig. 6:**
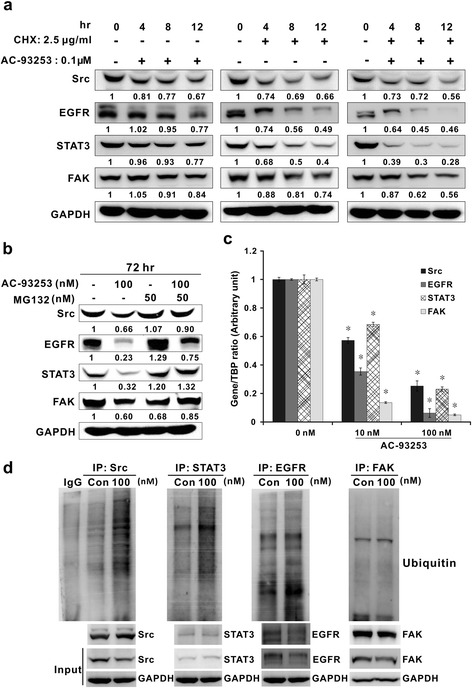
AC-93253 iodide inhibited Src and Src-related gene transcription and induced protein degradation. **a** AC-93253 iodide induced increases in protein degradation. Western blot analyses of Src, EGFR, STAT3, and FAK expression levels in PC9 cells treated with or without the protein synthesis inhibitor CHX and/or AC-93253 iodide for 4, 8, and 12 h. **b** AC-93253 iodide induced increases in ubiquitination, as determined by western blotting. The proteasome inhibitor MG132 and/or AC-93253 iodide were administered to PC9 cells for 72 h. GAPDH served as a loading control. **c** Repressive effects of AC-93253 iodide on Src, EGFR, STAT3, and FAK transcription as determined by real-time RT-PCR in PC9 cells. Relative gene expression levels were calculated using the comparative CT method (2^–ΔΔCT^). TBP: internal control. Each experiment was performed independently and in triplicate. *P < 0.05 compared with control (0 nM: 0.1% DMSO). **d** Enhanced ubiquitination of Src-related proteins induced by AC-93253 iodide. PC9 cell lysates with or without AC-93253 treatment were immunoprecipitated by the indicated antibodies and then analyzed by western blotting with anti-ubiquitin. GAPDH served as the internal control. Con represents 0 nM (0.1% DMSO)

The western blotting results showed that the expression levels of Src and its related proteins were lower in treated cells than in control cells, suggesting that AC-93253 iodide may cause protein degradation. Src, EGFR, STAT3, and FAK protein expression levels decreased in AC-93253 iodide-treated cells compared with control cells (Fig. 6a, left panel), and similar results were observed in cells treated with CHX (Fig. 6a, middle panel). Furthermore, in the PC9 cell line, degradation of the indicated proteins was significantly enhanced by co-treatment with CHX and AC-93253 iodide compared with other treatments (Fig. 6a, right panel), implying that AC-93253 iodide can enhance protein degradation.

To investigate whether the ubiquitin-proteasome system plays a role in AC-93253 iodide-induced protein degradation, we used the 26S proteasome inhibitor MG132 to inhibit protein ubiquitination. We found that co-treatment with AC-93253 iodide and MG132 can at least partially restore Src, EGFR, STAT3, and FAK expression levels compared with treatment with AC-93253 iodide alone (Fig. 6b). In addition, the real-time RT-PCR results showed that the mRNA expression levels of these genes were significantly downregulated by AC-93253 iodide even when the drug was administered at a relatively low concentration (Fig. 6c). Furthermore, to investigate whether AC-93253 iodide could enhance the ubiquitination of Src-related proteins, the immunoprecipitation of Src-related proteins and anti-ubiquitin western blotting were performed on PC9 cells treated with AC-93253 iodide. AC-93253 iodide could enhance the ubiquitination of Src, EGFR, and STAT3 but not of FAK in PC9 cells (Fig. 6d).

## Discussion

The first proto-oncogenic protein described [29] c-Src is widely expressed in many cell types and has been demonstrated to be involved in multiple signaling pathways that regulate cell growth and metastasis [30]. Its activity or expression has also been reported to contribute to drug resistance in patients with cancer and is associated with poor prognoses in such patients [31]. Computer-aided drug design (CADD) is a state-of-art technology that facilitates the efficient discovery and/or development of novel compounds, significantly shortens the R&D cycle, and decreases drug development-related costs [32]. In this study, using virtual molecular docking, we found that AC-93253 iodide is a potential Src inhibitor that can significantly inhibit cell function in vitro and NSCLC cell tumourigenesis in vivo. Moreover, we determined that AC-93253 iodide has synergistic effects when used in combination with gefitinib.

Only a few studies on AC-93253 iodide have been published; therefore, little is known about the compound. AC-93253 iodide functions like retinoid acid (RA) and can therefore act as an RA receptor-α (RAR-α) agonist [33]. RA has been shown to have anti-proliferative effects, and it induces apoptosis of breast cancer cells (MCF7) [34] and RAR-β expression, leading to growth arrest and apoptosis [35]. Furthermore, the results of previous studies indicated that AC-93253 is a novel inhibitor of the class III histone deacetylase SIRT2 and can selectively induce cytotoxicity in cancer cells [36] and affect the expression of a variety of genes important for the acquisition of chemoresistance and the progression of disease in melanoma cells [37]. However, the functional role of AC-93253 iodide in cancer as well as the mechanism underlying its anti-tumor activity is largely unknown. Here, we elucidated the multi-faceted role of AC-93253 iodide in cancer and the signaling pathways in which it may be involved. Additionally, to demonstrate that the inhibitory effects of AC-93253 iodide were specific to its molecular structure rather than to iodide itself, sodium iodide was used to treat PC9 and PC9/gef cells. Sodium iodide had no inhibitory effect on cell viability (Additional file 1: Figure S7) and on Src-related signaling (Additional file 1: Figure S8). These data revealed that the inhibitory effects of AC-93253 iodide were due to its specific molecular structure and not to iodide itself. To our knowledge, this is the first study to show that AC-93253 iodide can inhibit NSCLC progression by regulating multiple Src-related signaling pathways.

EGFR is frequently overexpressed in approximately 40–80% of NSCLC tumors; therefore, EGFR activity and/or expression is an important factor in the treatment of lung cancer that must be taken into consideration by clinicians attempting to manage the disease [38]. EGFR-activating mutations can trigger downstream signaling pathways, leading to uncontrolled cell proliferation [39]. Similar to EGFR, c-Src is also overexpressed in many types of cancer and has been shown to be co-overexpressed with EGFR in a subset of breast tumors [40]. A previous study showed that inhibiting Src activity may inhibit EGFR downstream signaling pathways, thereby inducing cancer cell apoptosis [17]. Therefore, Src can serve as a therapeutic target through which NSCLC treatment can be enhanced [15]. The Src inhibitor dasatinib has been approved for clinical use in patients with chronic myeloid leukemia (CML) [41] and can improve the efficacy of cisplatin in NSCLC cell lines when used in combination with it [42]. Moreover, dasatinib was recently shown to be a multi-kinase inhibitor that targets c-Kit, PDGFR, and FAK [43, 44]. However, like gefitinib, dasatinib is unable to inhibit the growth of NSCLC cells with wild-type EGFR (A549) or a T790M mutation (H1975) [17]. In contrast to dasatinib, AC-93253 iodide exerted cytotoxic effects on all the NSCLC cell lines tested, including the A549, PC9, PC9/gef, and H1975 cell lines, irrespective of their EGFR status. Furthermore, AC-93253 iodide was relatively less toxic to BEAS2B cells than to cancer cells in this study.

Previous reports have shown that Src can interact directly with EGFR [45, 46], leading to mutual phosphorylation and activation [46, 47]. Therefore, Src-EGFR cooperativity and interaction are critical for EGFR-mediated oncogenesis in NSCLC. Because of the crosstalk between Src and EGFR, inhibiting the activity of both proteins is feasible and may facilitate the successful treatment of NSCLC patients without EGFR-activating mutations or with acquired resistance-inducing EGFR mutations. Previous studies showed that c-Src kinase activity inhibition sensitizes cancer cells to EGFR inhibitors in epidermoid carcinoma cell lines and NSCLC cell lines [48, 49]. Thus, several clinical trials assessing the effects of combination therapy with dasatinib and EGFR TKIs (erlotinib and gefitinib) on NSCLC have been performed within the past few years [50, 51]. However, these phase II clinical trials did not produce the desired results with respect to investigations of the effects of those agents on NSCLC cells with acquired resistance-inducing EGFR mutations or without EGFR-activating mutations [52]. Our data indicated that AC-93253 iodide significantly sensitized gefitinib-resistant lung adenocarcinoma cells (A549, PC9/gef, and H1975) to gefitinib treatment in vitro, suggesting that the compound may reduce gefitinib doses, enhance gefitinib efficacy, and decrease targeted therapy costs and patient loads. These findings indicate that AC-93253 iodide may be a new candidate compound that can be used in place of dasatinib in combination therapy regimens comprising one of the two kinase inhibitors.

Src plays a pivotal role in many signaling pathways to promote cancer cell motility, survival, tumourigenesis, angiogenesis, and metastasis [53]. Among these pathways are some key pathways that regulate cancer progression, including the PI3K/AKT, STAT3, MEK/ERK, JNK, FAK, Paxillin, and p130cas pathways [14]. PI3K/Akt-related pathways activated by receptor tyrosine kinases (RTKs) and Src play key roles in mediating cell survival and regulating cell cycle progression [54]. Our data show that both Src and EGFR activity as well as PI3K phosphorylation and expression can be inhibited by AC-93253 iodide in EGFR mutant (PC9 and PC9/gef) and wild-type (A549) cells. It is well known that FAK-Src signaling can modulate actin cytoskeletal reorganization by activating its downstream substrates, including Paxillin, ERK, and p130cas, thereby facilitating cell migration [55]. Furthermore, JNK is the transcriptional regulator of matrix metalloproteinase (MMP)-2 and MMP-9; thus, JNK activation can result in proteolysis and increased cell invasion [56]. Our data revealed that AC-93253 iodide significantly represses FAK, JNK, Paxillin, and p130cas phosphorylation or protein expression in PC9, PC9/gef, and A549 cells, which may lead to cancer cell invasion and migration inhibition. A previous study showed that Src, RTKs, and integrins can activate pathways in which MEK and ERK may be involved, thereby inducing cell proliferation [57]. In this study, we showed that AC-93253 iodide could decrease MEK and ERK phosphorylation levels when administered at higher doses in the indicated cell lines.

Additionally, we found that the ability of AC-93253 iodide to decrease mRNA expression levels and increase protein degradation may be attributed to its effects on the ubiquitin-proteasome pathway, effects that are often observed with anti-tumor drugs [58]. Our data also demonstrated that the ubiquitination of Src-related proteins was increased by AC-93253 iodide. However, the degradation of FAK might involve mechanisms other than ubiquitination. While little is known about the role of AC-93253 iodide in transcriptional regulation, two previously published articles hinted that AC-93253 iodide may possess the ability to alter transcription activity. A cell-based reporter gene assay showed AC-93253 iodide to be a potent agonist of RAR-α, a nuclear receptor that can act as a transcription factor [59] in HEK cells [33]. Thus, stimulation by AC-93253 iodide might alter the transcriptional properties of RAR-α, leading to the direct or indirect transcriptional repression or activation of target genes. Most importantly, AC-93253 iodide has been reported to be a selective inhibitor of the deacetylase SIRT2 [36], which can reduce acetylation of the transcriptional co-activator p300 [60]. In general, histone deacetylase inhibitors can induce the hyperacetylation of histones [61] and the formation of open chromatin [62], resulting in either the up- or downregulation of genes. A previous study demonstrated that AC-93253 could downregulate the mRNA expression of oncogenes, apoptosis-related genes, and cell cycle control genes in melanoma cells [37]. Therefore, we speculated that the effect of AC-93253 on mRNA expression involves these mechanisms, especially in epigenetic regulation. Additional studies will be required to elucidate the detailed mechanisms underlying these phenomena.

Taken together, our findings indicate that AC-93253 iodide may directly or indirectly affect the expression of Src as well as that of downstream or related proteins and thus inhibit cancer progression. However, we cannot exclude the possibility that AC-93253 iodide may affect multiple targets. Polypharmacology is based on the idea that drugs affecting multiple targets can elicit various physiological responses [63]. It is believed that drugs that affect multiple targets can treat diseases more effectively than drugs affecting individual targets, regardless of whether these multi-target drugs are used alone or in combination with other agents [64], and that studies aiming to identify such drugs may represent a new direction for future drug discovery. For example, Sorafenib, a VEGFR, PDGFR, KIT, FLT3, and RAF inhibitor, was recently evaluated in clinical trials for its effectiveness against gastrointestinal stromal tumors [65]. Moreover, anlotinib, a multi-target TKI that can inhibit VEGFR2/3, FGFR1-4, PDGFR α/β, c-Kit, and Ret [66], was evaluated in pre-clinical trials in patients with advanced solid tumors.

## Conclusions

In summary, we found that AC-93253 iodide may have multi-target-related inhibitory effects on NSCLC and may have synergistic effects when used in combination with gefitinib. These results indicate that AC-93253 iodide may be useful as a treatment for cancer. Therefore, whether used alone or in combination with other drugs, AC-93253 iodide may be a compound on which future therapies can be based and should thus be evaluated in future drug discovery and development studies.

## Additional file

Additional file 1: Table S1. The 15 compounds predicted to bind open-form Src by Discovery Studio Client, based on the strengths of their interactions with the protein. **Table S2.** The combination index (CI) and fractional effect (FE) of combination treatment with AC-93253 iodide and gefitinib (Iressa) in lung cancer A549 cells. **Table S3.** The CI and FE of combination treatment with AC-93253 iodide and gefitinib (Iressa) in lung cancer PC9/gef cells. **Table S4.** The CI and FE of combination treatment with AC-93253 iodide and gefitinib (Iressa) in lung cancer H1975 cells. **Figure S1.** Suppression of Src by candidate compounds in PC9 cell line. **Figure S2.** Cytotoxic effects of AC-93253 iodide on non-tumour BEAS2B cells, as determined by cell viability assay. **Figure S3.** Western blotting for the effects of AC-93253 iodide on the expression of Src and its associated proteins in the A549 cell line. **Figure S4.** AC-93253 iodide-mediated suppression of anchorage-dependent growth in A549 cells. **Figure S5.** The effects of AC-93253 iodide treatment on CL1-5 cells, as determined by anchorage-independent growth assay. **Figure S6.** Phosphorylation and expression of Src-related proteins in gefitinib-resistant lung adenocarcinoma cells treated with AC93253 iodide and gefitinib, separately and in combination. **Figure S7.** Cytotoxic effect of sodium iodide on PC9 and PC9/gef cell lines. **Figure S8.** Western blotting for the effects of sodium iodide on the phosphorylation and expression of Src and its related proteins in the PC9 and PC9/gef cells. (DOCX 2294 kb)
