# Supplementary material for: AC-93253 iodide, a novel Src inhibitor, suppresses NSCLC progression by modulating multiple Src-related signaling pathways
Source: J Hematol Oncol. 2017 Nov 13;10:172. doi: 10.1186/s13045-017-0539-3 (PMC5683468; doi:10.1186/s13045-017-0539-3)
Supplement: Additional file 1: Table S1. — The 15 compounds predicted to bind open-form Src by Discovery Studio Client, based on the strengths of their interactions with the protein. Table S2. The combination index (CI) and fractional effect (FE) of combination treatment with AC-93253 iodide and gefitinib (Iressa) in lung cancer A549 cells. Table S3. The CI and FE of combination treatment with AC-93253 iodide and gefitinib (Iressa) in lung cancer PC9/gef cells. Table S4. The CI and FE of combination treatment with AC-93253 iodide and gefitinib (Iressa) in lung cancer H1975 cells. Figure S1. Suppression of Src by candidate compounds in PC9 cell line. Figure S2. Cytotoxic effects of AC-93253 iodide on non-tumour BEAS2B cells, as determined by cell viability assay. Figure S3. Western blotting for the effects of AC-93253 iodide on the expression of Src and its associated proteins in the A549 cell line. Figure S4. AC-93253 iodide-mediated suppression of anchorage-dependent growth in A549 cells. Figure S5. The effects of AC-93253 iodide treatment on CL1-5 cells, as determined by anchorage-independent growth assay. Figure S6. Phosphorylation and expression of Src-related proteins in gefitinib-resistant lung adenocarcinoma cells treated with AC93253 iodide and gefitinib, separately and in combination. Figure S7. Cytotoxic effect of sodium iodide on PC9 and PC9/gef cell lines. Figure S8. Western blotting for the effects of sodium iodide on the phosphorylation and expression of Src and its related proteins in the PC9 and PC9/gef cells. (DOCX 2294 kb) [file 13045_2017_539_MOESM1_ESM.docx]

**Additional file 1**

**AC-93253 iodide, a novel Src inhibitor, suppresses NSCLC progression by modulating multiple Src-related signalling pathways**

Yi-Hua Lai, Sih-Yin Lin, Yu-Shan Wu, Huei-Wen Chen, and Jeremy J.W. Chen

***Correspondence:** Jeremy J.W. Chen, Institute of Biomedical Sciences, National Chung Hsing University, No. 145, Xingda Rd., South Dist., Taichung 40227, Taiwan, ROC. Phone: 886-4-22840896 ext. 125; Fax: 886-4-22853469; Email: [jwchen@dragon.nchu.edu.tw](mailto:jwchen@dragon.nchu.edu.tw)

**Table S1.** The 15 compounds predicted to bind open-form Src by Discovery Studio Client, based on the strengths of their interactions with the protein.

|  | **LOPAC ID** | **Drug name** | **MW** | **Libdock score** | **Interaction** |
| --- | --- | --- | --- | --- | --- |
|  |  |  |  |  | **force** |
| **L1** | **11G07** | Niclosamide | 327.12 | 54.01 | π-π |
| **L2** | **13C06** | 1,10-Phenanthroline monohydrate | 180.21 | 68.27 | π-σ |
| **L3** | **5H06** | P1,P4-Di(adenosine-5')tetraphosphate triammonium | 887.48 | 128.72 | H bond |
| **L4** | **3H08** | Supercinnamaldehyde | 201.22 | 38.86 | π-σ |
| **L5** | **1D05** | Aminopterin | 440.42 | 103.99 | π-σ |
| **L6** | **10H02** | L-745,870 hydrochloride | 363.29 | 83.30 | π-σ |
| **L7** | **14A09** | '6-Nitrobenzo[b]thiophene-1,1-dioxide (Stattic) | 211.19 | 72.19 | H bond |
| **L8** | **14F11** | Salmeterol xinafoate | 603.76 | 136.07 | H bond |
| **L9** | **5B09** | Dequalinium chloride hydrate | 527.58 | 133.86 | π-+ |
| **L10** | **6H06** | AC-93253 iodide | 488.43 | 21.45 | π-+ |
| **L11** | **15G09** | Thio-NADP sodium | 781.46 | 128.25 | H bond/π-+ |
| **L12** | **13D08** | SID7969543 | 452.46 | 122.51 | H bond |
| **L13** | **5A04** | CGS-21680 hydrochloride | 535.99 | 124.25 | H bond |
| **L14** | **7B09** | Formoterol | 804.90 | 127.13 | π-σ |
| **L15** | **10C09** | Mibefradil dihydrochloride | 568.56 | 120.41 | H bond |

**Table S2.** The combination index (CI) and fractional effect (FE) of combination treatment with AC-93253 iodide and gefitinib (Iressa) in lung cancer A549 cells.

| **Gefitinib**  **(μM)** | **AC-93253 iodide (μM)** | | | | | | | | | | |
| --- | --- | --- | --- | --- | --- | --- | --- | --- | --- | --- | --- |
|  | **0.01** | | |  | **0.05** | | |  | **0.1** | | |
|  | FE |  | CI |  | FE |  | CI |  | FE |  | CI |
| **0.01** | 0.233±0.039 |  | 0.469±0.177 |  | 0.364 ±0.130 |  | 0.838±0.441 |  | 0.867 ± 0.034 |  | 0.327±0.060 |
| **0.05** | 0.304±0.071 |  | 0.391±0.166 |  | 0.551 ±0.002 |  | 0.474±0.066 |  | 0.826±0.036 |  | 0.400±0.052 |
| **0.1** | 0.440±0.015 |  | 0.259±0.032 |  | 0.371 ±0.013 |  | 0.754±0.121 |  | 0.788±0.038 |  | 0.468±0.047 |
| **0.25** | 0.423±0.044 |  | 0.301±0.081 |  | 0.489 ±0.024 |  | 0.571±0.047 |  | 0.775±0.071 |  | 0.495±0.110 |
| **0.5** | 0.328±0.037 |  | 0.445±0.146 |  | 0.591 ±0.069 |  | 0.479±0.121 |  | 0.779±0.096 |  | 0.495±0.168 |

**Table S3.** The CI and FE of combination treatment with AC-93253 iodide and gefitinib (Iressa) in lung cancer PC9/gef cells.

| **Gefitinib**  **(μM)** | **AC-93253 iodide (μM)** | | | | | | | | | | |
| --- | --- | --- | --- | --- | --- | --- | --- | --- | --- | --- | --- |
|  | **0.01** | | |  | **0.05** | | |  | **0.1** | | |
|  | FE |  | CI |  | FE |  | CI |  | FE |  | CI |
| **0.01** | 0.058±0.067 |  | 1.844±1.520 |  | 0.619 ±0.053 |  | 0.756±0.064 |  | 0.922±0.044 |  | 0.646±0.072 |
| **0.05** | 0.034±0.034 |  | 1.964±1.364 |  | 0.660 ±0.043 |  | 0.707±0.066 |  | 0.937±0.032 |  | 0.593±0.053 |
| **0.1** | 0.017±0.009 |  | 2.210±1.032 |  | 0.706 ±0.030 |  | 0.645±0.072 |  | 0.939±0.049 |  | 0.563±0.141 |
| **0.25** | 0.037±0.038 |  | 1.985±1.397 |  | 0.765 ±0.074 |  | 0.584±0.003 |  | 0.990±0.013 |  | 0.237±0.158 |
| **0.5** | 0.107±0.135 |  | 1.772±1.616 |  | 0.763 ±0.079 |  | 0.610±0.009 |  | 0.989±0.015 |  | 0.259±0.173 |

**Table S4.** The CI and FE of combination treatment with AC-93253 iodide and gefitinib (Iressa) in lung cancer H1975 cells.

| **Gefitinib**  **(μM)** | **AC-93253 iodide (μM)** | | | | | | | | | | |
| --- | --- | --- | --- | --- | --- | --- | --- | --- | --- | --- | --- |
|  | **0.01** | | |  | **0.05** | | |  | **0.1** | | |
|  | FE |  | CI |  | FE |  | CI |  | FE |  | CI |
| **0.01** | 0.010±0.010 |  | 0.786±0.248 |  | 0.198±0.093 |  | 0.841±0.223 |  | 0.999±0.010 |  | 0.174±0.081 |
| **0.05** | 0.010±0.010 |  | 0.799±0.245 |  | 0.334±0.158 |  | 0.698±0.173 |  | 0.919±0.099 |  | 0.498±0.342 |
| **0.1** | 0.072±0.087 |  | 0.699±0.449 |  | 0.427±0.199 |  | 0.633±0.156 |  | 0.921±0.111 |  | 0.437±0.448 |
| **0.25** | 0.044±0.048 |  | 0.734±0.417 |  | 0.414±0.013 |  | 0.634±0.020 |  | 0.955±0.007 |  | 0.493±0.124 |
| **0.5** | 0.170±0.082 |  | 0.449±0.094 |  | 0.537±0.063 |  | 0.584±0.052 |  | 0.933±0.085 |  | 0.496±0.363 |

**
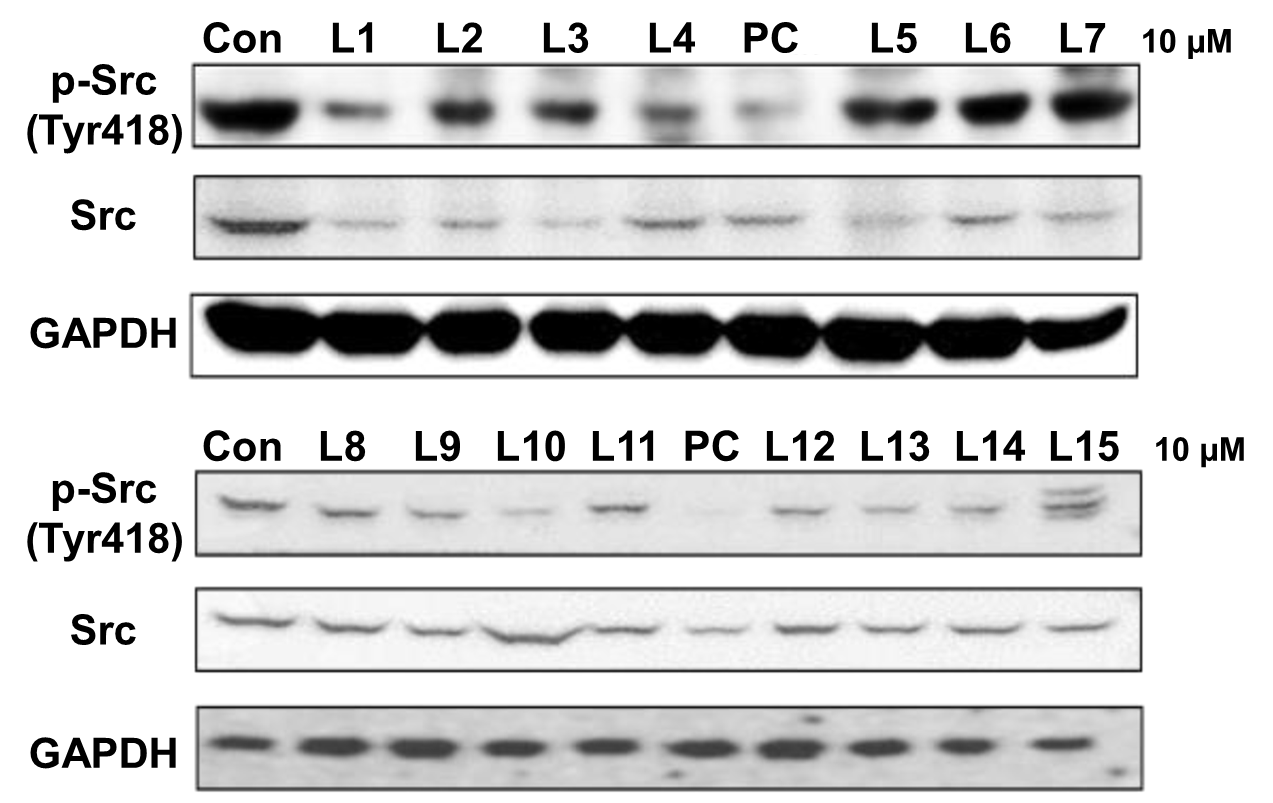
**

**Figure S1.** Suppression of Src by candidate compounds in PC9 cell line. Western blotting of Src phosphorylation and expression in PC9 cells at 24 hours after candidate compounds treatment. Con indicates 0.1% DMSO; PC indicates 100 nM dasatinib as a positive control; and GAPDH was used as an internal control. Each experiment was independently performed in triplicate.

**
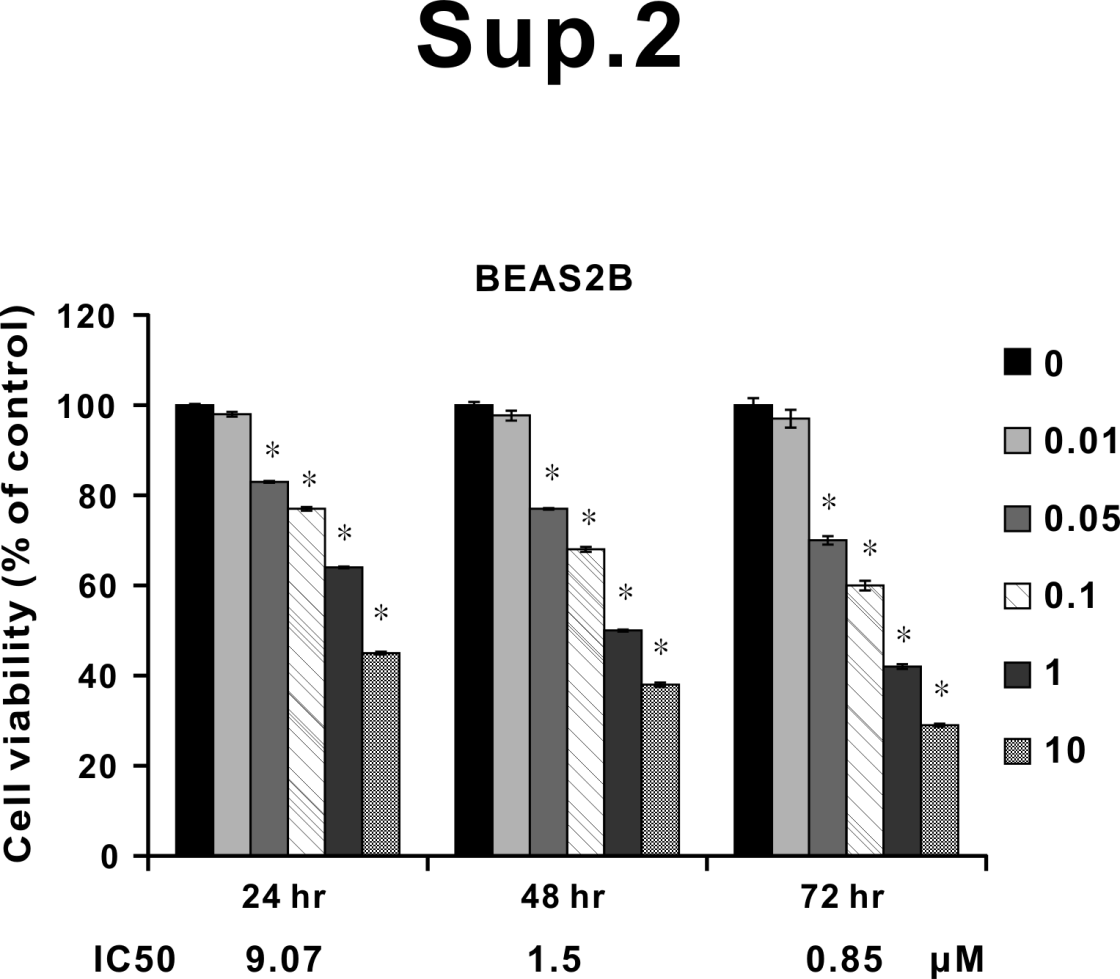
**

**Figure S2.** Cytotoxic effects of AC-93253 iodide on non-tumour BEAS2B cells, as determined by cell viability assay. PrestoBlue cell viability assay was performed to investigate the cytotoxic effects of AC-93253 iodide on BEAS2B cells at 24, 48, or 72 hours after treatment. The IC50 values at 24, 48, and 72 hours were 9.07, 1.5, and 0.85 μM, respectively. Each experiment was performed independently and in triplicate; 0 μM; 0.1% DMSO. *P<0.05 compared with control (0 μM).


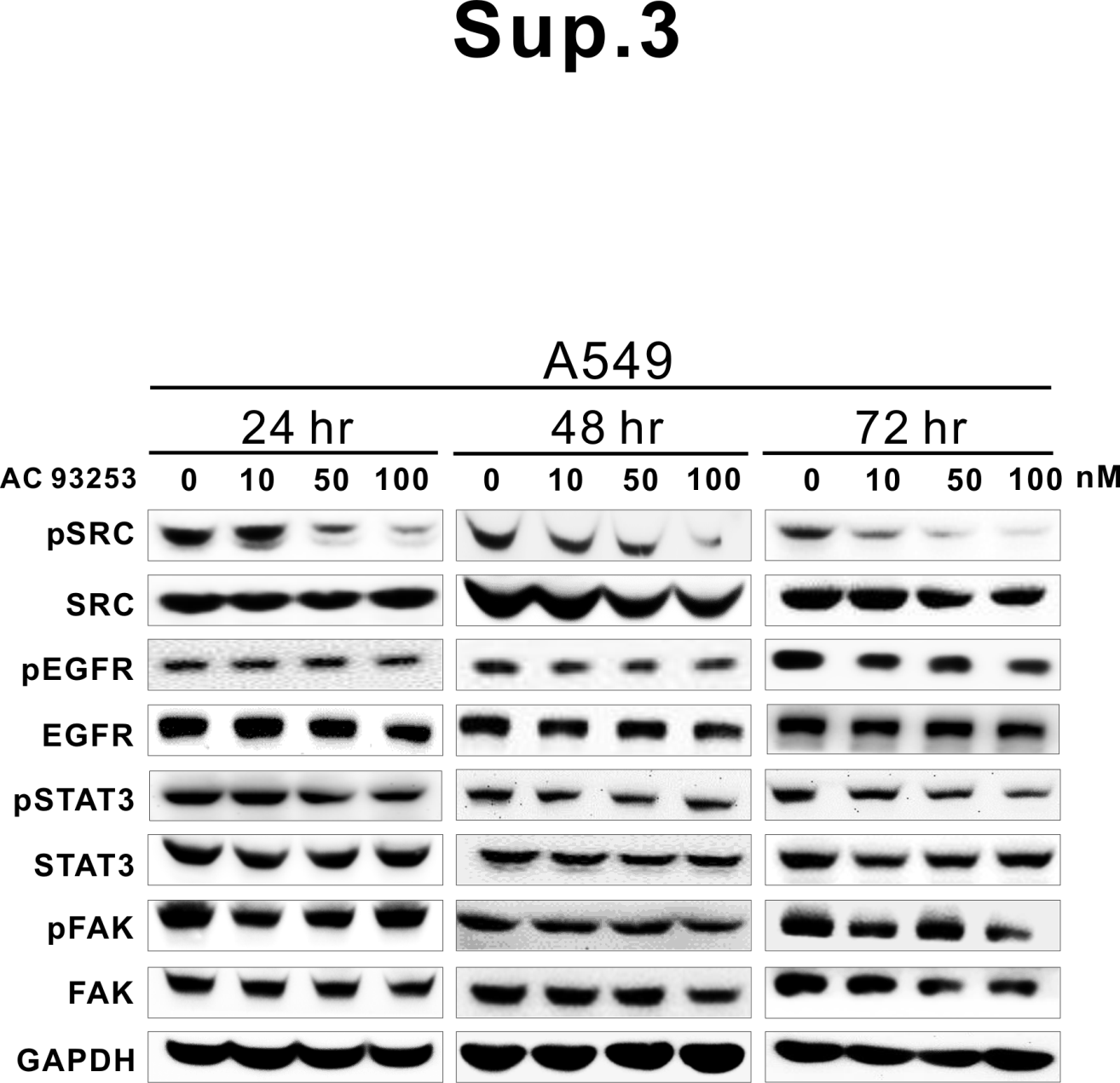


**Figure S3.** Western blotting for the effects of AC-93253 iodide on the expression of Src and its associated proteins in the A549 cell line. A549 cells were treated with AC-93253 iodide at the indicated concentrations and then subjected to western blot analyses. GAPDH served as an internal control. Each experiment was performed independently and in triplicate; 0 nM; 0.1% DMSO.


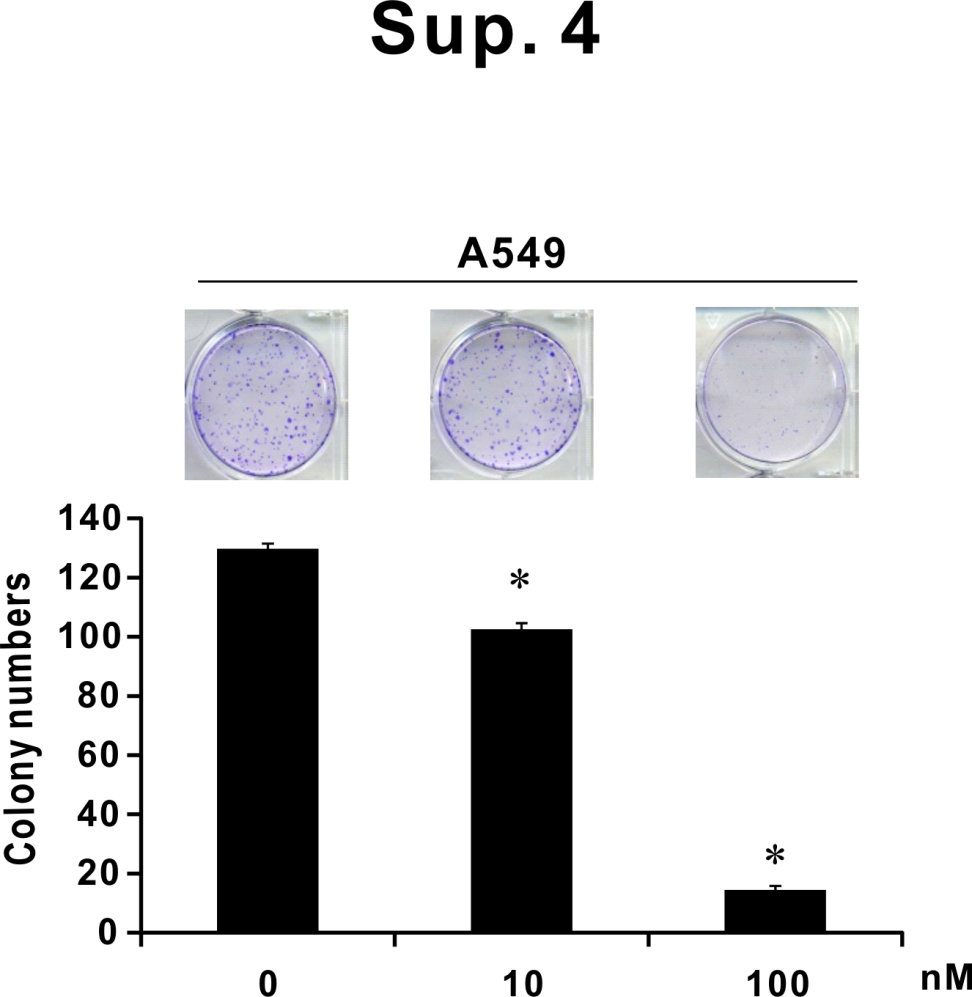


**Figure S4.** AC-93253 iodide-mediated suppression of anchorage-dependent growth in A549 cells. The cells were grown in a culture dish and were treated with various concentrations of AC-93253 iodide. The colonies with a diameter ≥0.3 mm were selected. Each experiment was performed independently and in triplicate; 0 nM represents 0.1% DMSO. *P<0.05 compared with vehicle-treated control (0 nM, 0.1% DMSO).


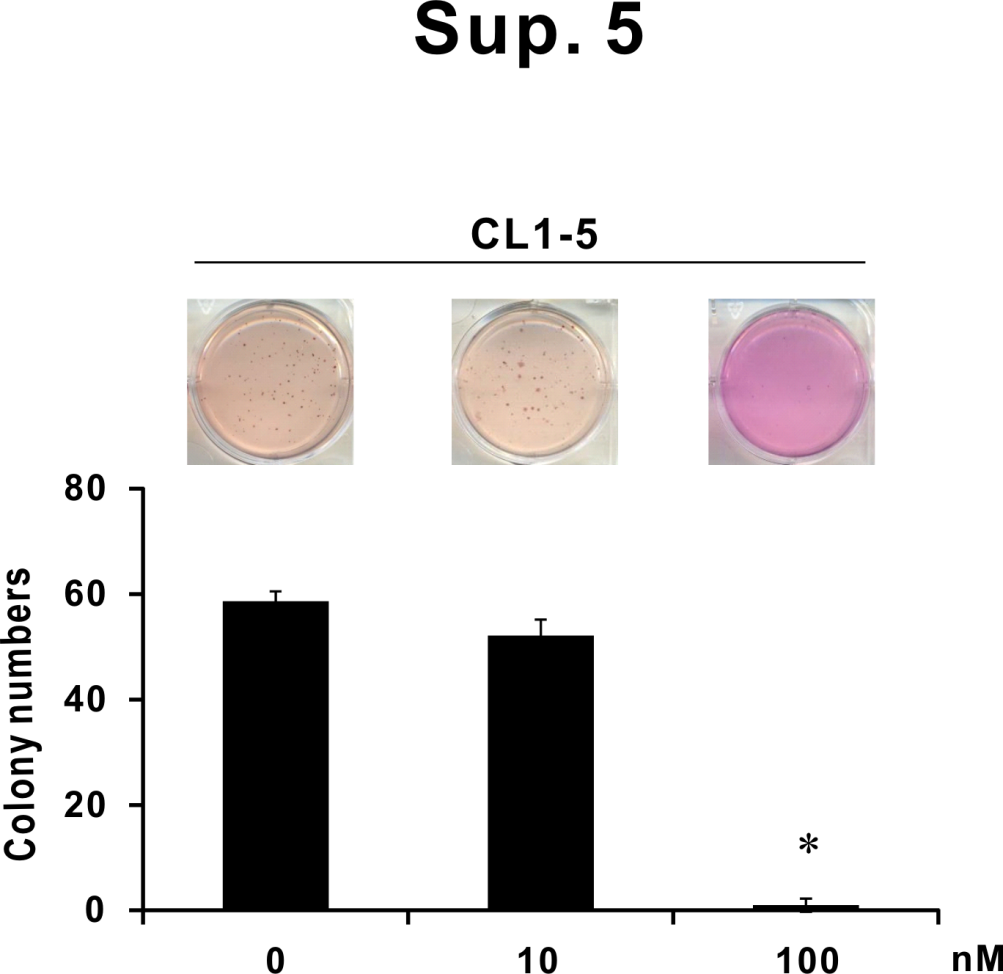


**Figure S5.** The effects of AC-93253 iodide treatment on CL1-5 cells, as determined by anchorage-independent growth assay. The cells grown in soft agar were treated with AC-93253 iodide and then evaluated by clonogenic assays. The colonies with diameters ≥0.5 mm were counted. Each experiment was performed independently and in triplicate; 0 nM represents 0.1% DMSO. *P<0.05 compared with vehicle-treated control (0 nM).


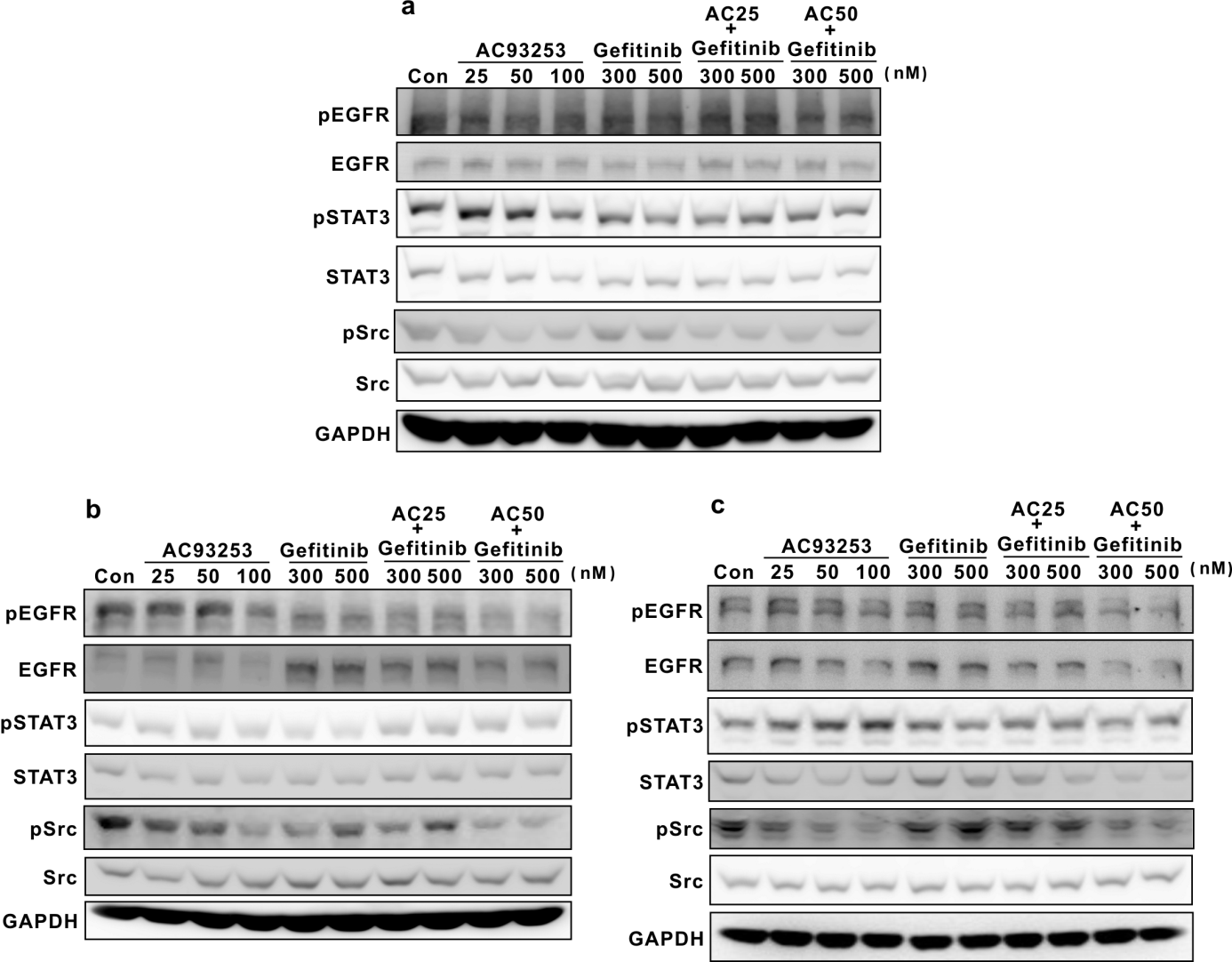


**Figure S6.** Phosphorylation and expression of Src-related proteins in gefitinib-resistant lung adenocarcinoma cells treated with AC-93253 iodid and gefitinib, separately and in combination. The indicated combinations of AC-93253 iodide and gefitinib were used to treat lung adenocarcinoma A549 cells (a), PC9/gef cells (b), and H1975 cells (c) for 72 hours; the cells were then analysed by western blot. Con represents 0 nM (0.1% DMSO). GAPDH served as a loading control. Each experiment was performed independently and in triplicate.

**
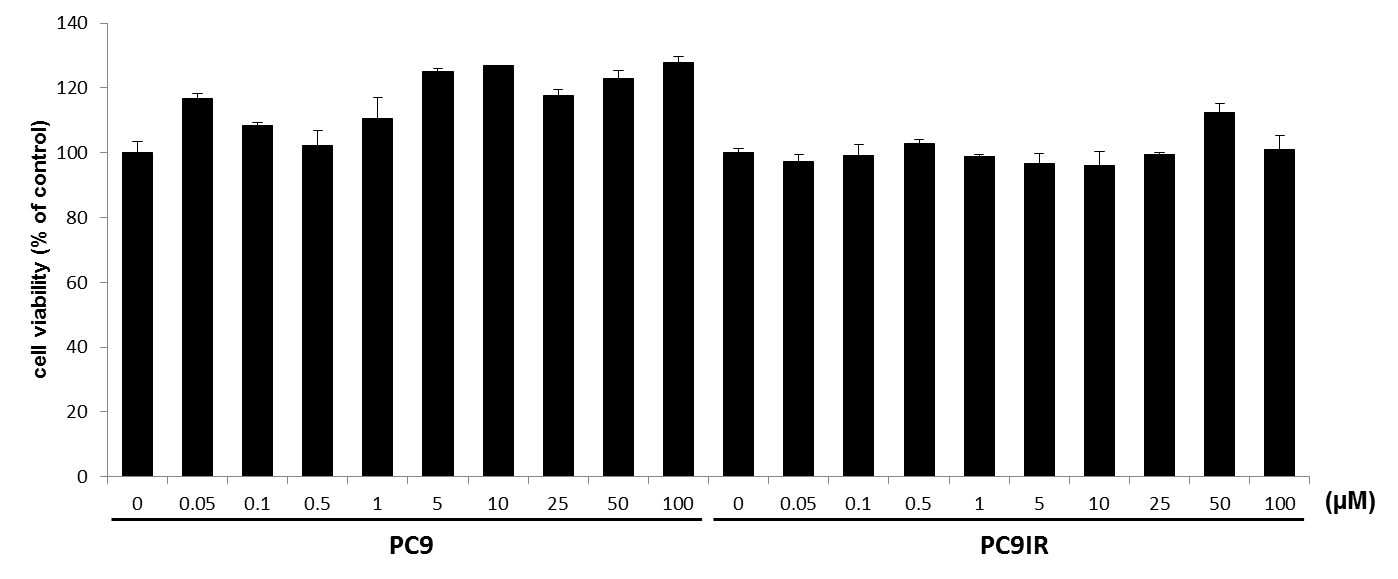
**

**Figure S7.** Cytotoxic effect of sodium iodide on PC9 and PC9/gef cell lines. PrestoBlue cell viability assays were performed to investigate the cytotoxic effects of sodium iodide on these cell lines after treatment for 72 hours. The results are shown as percentages of the control response (0 μM), and 0 μM represents 0.1% DMSO. Each treatment was independently performed in triplicate.

**
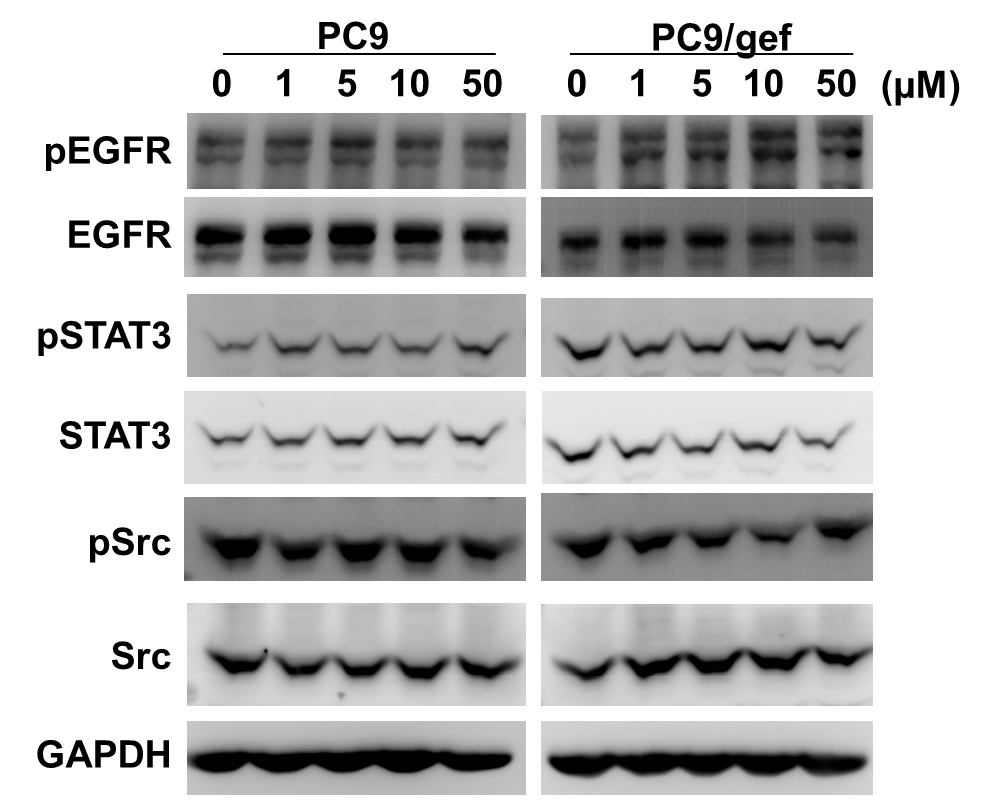
**

**Figure S8.** Western blotting for the effects of sodium iodide on the phosphorylation and expression of Src and its related proteins in the PC9 and PC9/gef cells. Cells were treated with sodium iodide at the indicated concentrations and then subjected to western blot analyses. GAPDH served as an internal control. Each experiment was performed independently and in triplicate; 0 nM: 0.1% DMSO.
